# Exploring the inverse relationship between serum total bilirubin and systemic immune-inflammation index: insights from NHANES data (2009–2018)

**DOI:** 10.1186/s40001-024-01963-9

**Published:** 2024-07-12

**Authors:** Shan-Shan Huang, Yi Ding, Xiao-Na Yi, Hai-Yan Mao, Zhen-Ye Xie, Xing-Kai Shen, Yan Lu, Jing Yan, You-Wei Wang, Zhou-Xin Yang

**Affiliations:** 1 Department of Critical Care Medicine, Ningbo Medical Center Lihuili Hospital, Ningbo, 315100 China; 2 Zhejiang Key Laboratory of Geriatrics and Geriatrics Institute of Zhejiang Province, Zhejiang Hospital, Hangzhou, 310030 China; 3 Institute of Medical Engineering & Translational Medicine, Tianjin University, Tianjin, 300072 China

**Keywords:** Total bilirubin, Systemic immune-inflammation index, Relationship, NHANES

## Abstract

**Background:**

Bilirubin is known for its multifaceted attributes, including antioxidant, anti-inflammatory, immunomodulatory, and antiapoptotic properties. The systemic immune-inflammation index (SII) is a recent marker that reflects the balance between inflammation and immune response. Despite the wealth of information available on bilirubin’s diverse functionalities, the potential correlation between the total bilirubin (TB) levels and SII has not been investigated so far.

**Methods:**

Leveraging data from the National Health and Nutrition Examination Survey spanning 2009–2018, the TB levels were categorized using tertiles. Employing the chi-squared test with Rao and Scott’s second-order correction and Spearman’s rank correlation analysis, the association between TB and SII was examined. The potential nonlinearities between TB and SII were evaluated using restricted cubic spline (RCS) analysis. Weighted linear regression, adjusted for covariates, was used to explore the correlation between TB and SII, with further subgroup analyses.

**Results:**

A total of 16,858 participants were included, and the findings revealed significant SII variations across TB tertiles (*p* < 0.001). The third tertile (Q3) exhibited the lowest SII level at 495.73 (295.00) 1000 cells/µL. Spearman rank correlation disclosed the negative association between TB and SII. RCS analysis exposed the lack of statistically significant variations in the nonlinear relationship (*p* > 0.05), thereby providing support for a linear relationship. Weighted linear regression analysis underscored the negative correlation between TB and SII (β 95% CI − 3.9 [− 5.0 to  − 2.9], *p* < 0.001). The increase in the TB levels is associated with a significant linear trend toward decreasing SII. After controlling for relative covariates, this negative correlation increased (*p* < 0.001). Subgroup analysis confirmed the significant negative TB–SII association**.**

**Conclusion:**

A notable negative correlation between TB and SII implies the potential protective effects of bilirubin in inflammation-related diseases.

## Background

Inflammation is a versatile and evolutionarily conserved physiological mechanism that responds to microbial invasion and triggers a preemptive program during tissue damage [1]. Inflammation serves as the foundation for various physiological and pathological processes and is regarded as the “common ground” for multifactorial diseases, such as chronic inflammatory rheumatic diseases, type 2 diabetes, cardiovascular diseases, neurodegenerative disorders, obesity, cancer, asthma, and aging [2–4].

Presently, various clinical indicators are used to characterize the acute or chronic inflammatory states associated with diverse diseases, including C-reactive protein, procalcitonin, white blood cell count, neutrophil ratio, interleukin (IL)-6, and tumor necrosis factor-α (TNF-α) [5, 6]. In 2014, the systemic immune-inflammation index (SII) was initially defined as “lymphocyte count × neutrophil count/platelet count.” This index indicates the delicate balance between inflammation and immune response. The elevated SII levels signify an augmented inflammatory state and a compromised immune response [7].

Current clinical research emphasizes the utility of SII as a predictive and evaluative marker for inflammatory diseases. For example, Liu et al. reported that SII was a potential indicator for predicting the severity of acute pancreatitis [8]. Furthermore, SII has been recognized as an independent prognostic marker in individuals with psoriasis and psoriatic arthritis [9]. Moreover, a past study investigated SII as an independent prognostic indicator in the context of psoriasis and psoriatic arthritis [10]. In addition, a noteworthy J-shaped association was observed between SII and periodontitis, implying the possibility of an auxiliary therapeutic strategy for periodontal disease [11].

Bilirubin, which is a part of the superfamily of tetrapyrrole compounds, is characterized by its insolubility in water. It has been considered as the ultimate toxic byproduct of heme catabolism in systemic circulation [12]. Bilirubin has been reported to be associated with infant neuronal damage, causing irreversible harm to the brain and central nervous system. Additionally, it is a marker for liver dysfunction [13–15]. An expanding body of evidence suggests that bilirubin transcends its conventional perception and has increasingly been recognized for its antioxidant, anti-inflammatory, immunomodulatory, and antiapoptotic properties [16].

In the context of anti-inflammatory investigations, bilirubin has been established to exert inhibitory effects on the upregulation of E-selectin, vascular cell adhesion molecule-1, and intercellular adhesion molecule-1 induced by TNF-α in vitro [17]. Furthermore, a past study have identified a negative correlation between serum bilirubin levels and the inflammatory marker C-reactive protein [18]. Nevertheless, the relationship between the total bilirubin levels and SII has not been extensively explored. Therefore, in this study, data from the National Health and Nutrition Examination Survey (NHANES) was leveraged to determine the correlation between the bilirubin levels and SII in adult participants.

## Methods

### Data source and study population

All data for this study was derived from NHANES conducted in the United States. NHANES employs a complex, multistage probability sampling method, providing extensive information on the nutrition and health of the general U.S. population. The data encompass demographic statistics, dietary information, examination results, laboratory findings, questionnaire responses, and restricted-access data. Details about the NHANES database, including study design and data, are publicly available at https://www.cdc.gov/nchs/nhanes/. The NHANES study was approved by the National Center for Health Statistics (NCHS) Research Ethics Review Board, and all survey participants signed informed consent forms. Detailed information about the NCHS Research Ethics Review Board approval can be accessed from the NHANES website (https://www.cdc.gov/nchs/nhanes/irba98.htm).

**Fig. 1:**
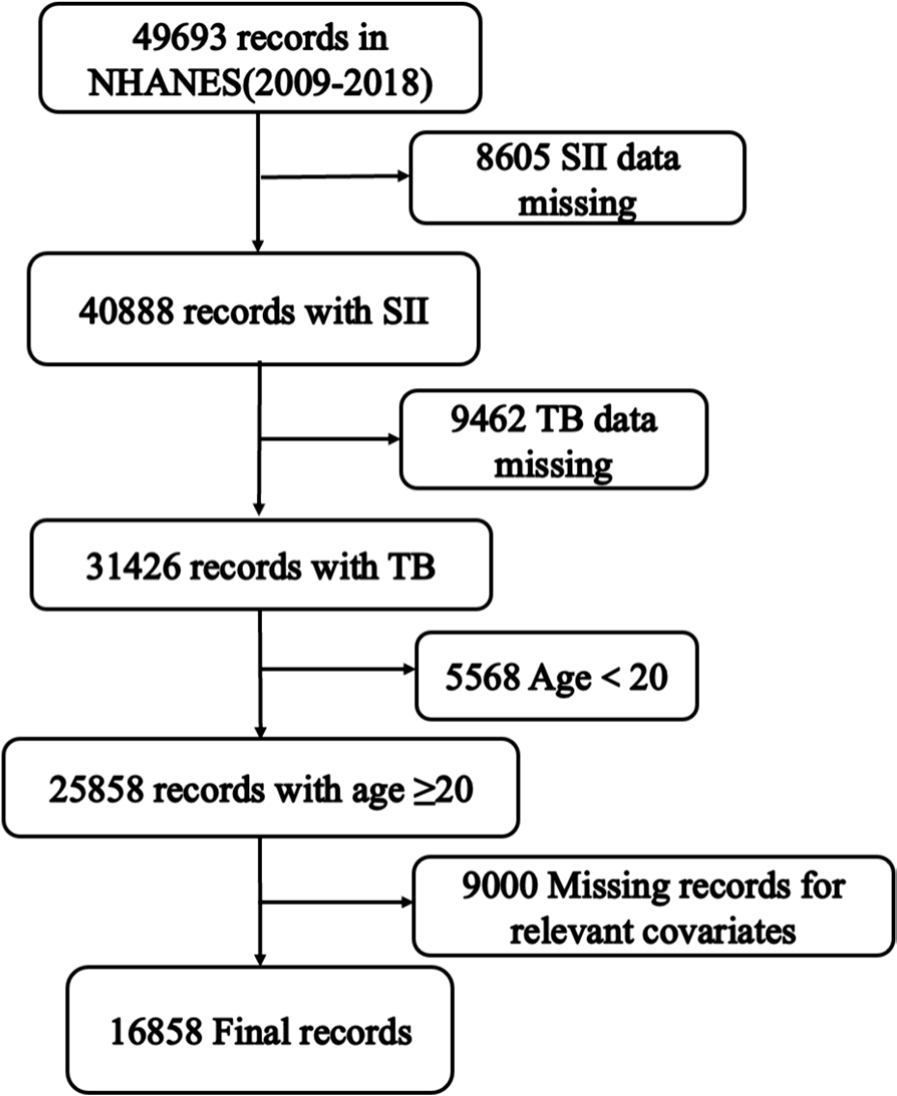
The selection process for this study (*SII* systemic immune-inflammation index, *TB* total bilirubin)

This study includes data from NHANES cycles spanning 2009–2018, covering a total of five consecutive periods and involving 49,693 participants. Figure 1 depicts the selection process for this study. The following participants were excluded: [1] 8605 individuals with missing SII data; [2] 9462 individuals with missing total bilirubin (TB) data; [3] 5568 participants under the age of 20; [4] 9000 participants with missing data for relevant variables. Ultimately, 16,858 participants were included in the final analysis.

### Measurement of TB and SII

TB was measured in the serum or plasma with the timed endpoint diazo method (Jendrassik-Grof) using DxC800. According to a previous study [7], the SII was calculated by multiplying the platelet count with the neutrophil count and dividing the value by the lymphocyte count. Whole blood cell counts were determined using the Beckman Coulter DxH 800 instrument at the NHANES Mobile Examination Center (MEC) and were expressed as × 1000 cells/µL.

### Study-related covariates

To minimize the potential impact of confounding factors, the following covariates were primarily included: age, sex (male and female), race (Hispanic, non-Hispanic white, non-Hispanic black, other/multiracial), the education level (less than high school, high school, and more than high school), body mass index (BMI, underweight, normal weight, overweight, and obese), physical activity (PA, < 600 MET, ≥ 600 MET, metabolic equivalent) [19, 20], smoking status (yes or no), drinking status (yes or no), white blood cell count (WBC), neutrophil count (Neu), platelet count (Plt), the level of creatinine, blood potassium level (K), and blood sodium (Na), hypertension (yes or no), and diabetes (yes or no).

### Statistical analysis

Weighted methodologies were employed to mitigate data volatility. Continuous variables were analyzed using the chi-squared test with Rao and Scott’s second-order correction, and the results were presented in terms of weighted means and standard deviations. Categorical variables were assessed using the Wilcoxon rank–sum test, with the outcomes expressed as weighted percentages. Initial normality testing involved the Kolmogorov–Smirnov test, followed by Spearman’s rank correlation analysis for variable relationships. Continuous variable TB was stratified into three quartiles. Weighted linear regression analysis was performed to examine the correlation between TB and SII, with subgroup findings depicted as forest plots. The following three models were utilized: Model 1 (unadjusted), Model 2 (adjusted for age, sex, race, education, creatinine, PA, and BMI), and Model 3 (age, sex, race, education, creatinine, PA, BMI, smoking status, drinking status, hypertension, and diabetes). All data analyses were performed using SPSS 26.0 and R 4.2.3. *p* < 0.05 was considered to indicate statistical significance.

## Results

### Weighted characteristics of the participants

This study included a total of 16,858 participants, with a weighted average age of 45.96 years. Women constituted 51.89%, and men accounted for 48.11% (Table 1). The weighted median SII was 517.19 (298.64) 1000 cells/µL. Significant differences in the SII levels were observed across different tertiles based on the TB levels (*p* < 0.001). Especially, the third tertile (Q3) exhibited the lowest level at 495.73 (295.00) 1000 cells/µL compared with the other two tertiles. Moreover, TB levels were found to be significantly linked to sex, race, BMI, education level, white blood cell count, lymphocyte count, neutrophil count, platelet count, the level of creatinine, PA, and potassium, smoking status, hypertension, and diabetes (*p* < 0.05). Nonetheless, the TB levels were not significantly correlated with age, blood sodium levels, and drinking status (*p* > 0.05).

**Table 1:** Weighted characteristics of the participants by tertiles of TB

Characteristic	Overall N = 16,858	TB Tertiles			*p*-value^2^
		Q1, N = 4546^1^	Q2, N = 5268^1^	Q3, N = 7044^1^	
SII (1000 cells/µl)	517.19 (298.64)	545.09 (307.33)	522.08 (293.51)	495.73 (295.00)	< 0.001
TB (μmol/L)	10.95 (5.43)	5.40 (1.55)	9.39 (0.85)	15.64 (4.90)	< 0.001
Sex					< 0.001
Female	48.11%	63.55%	53.23%	34.49%	
Male	51.89%	36.45%	46.77%	65.51%	
Age (year)	45.96 (16.52)	45.63 (16.49)	46.30 (16.28)	45.92 (16.69)	0.286
Race					< 0.001
Hispanic	13.87%	15.51%	14.85%	12.11%	
Non-hispanic white	10.06%	12.21%	10.82%	8.12%	
Non-hispanic black	68.15%	63.12%	66.98%	72.22%	
Other/multiracial	7.93%	9.15%	7.36%	7.55%	
Education					< 0.001
Less than high school	65.68%	62.45%	64.69%	68.46%	
High school	12.10%	12.11%	13.37%	11.17%	
More than high school	22.22%	25.43%	21.93%	20.37%	
BMI					< 0.001
Normal	28.95%	23.26%	28.53%	32.91%	
Obese	36.17%	45.95%	36.96%	29.30%	
Overweight	33.29%	29.31%	33.10%	35.98%	
Underweight	1.60%	1.48%	1.41%	1.81%	
PA					< 0.001
< 600 MET min/week	44.23%	44.85%	46.75%	42.02%	
≥ 600 MET min/week	55.77%	55.15%	53.25%	57.98%	
Smoker					0.016
Yes	43.38%	45.37%	44.18%	41.53%	
No	56.62%	54.63%	55.82%	58.47%	
Drinker					0.069
Yes	83.38%	83.71%	82.20%	84.01%	
No	16.62%	16.29%	17.80%	15.99%	
Creatinine (μmol/L)	77.93 (27.73)	73.74 (22.53)	76.90 (27.00)	81.35 (30.70)	< 0.001
Na (mmol/L)	139.40 (2.29)	139.57 (2.40)	139.34 (2.33)	139.33 (2.19)	0.371
K (mmol/L)	4.00 (0.33)	4.02 (0.35)	4.00 (0.33)	3.99 (0.33)	0.025
WBC (1000 cells/µl)	7.20 (2.30)	7.76 (2.68)	7.27 (2.25)	6.79 (1.96)	< 0.001
Neu (1000 cells/µl)	4.25 (1.65)	4.56 (1.75)	4.30 (1.65)	4.02 (1.54)	< 0.001
Plt (1000 cells/µl)	238.11 (58.99)	254.64 (63.54)	240.58 (57.06)	225.71 (54.30)	< 0.001
Lym (1000 cells/µl)	2.14 (1.16)	2.34 (1.63)	2.16 (1.13)	1.99 (0.72)	< 0.001
Hypertension					0.010
Yes	28.60%	31.09%	28.04%	27.41%	
No	71.40%	68.91%	71.96%	72.59%	
Diabetes					< 0.001
Yes	8.40%	10.04%	9.02%	6.91%	
No	91.60%	89.96%	90.98%	93.09%	

*TB* total bilirubin, *SII* systemic immune-inflammation index, *BMI* body mass index, *PA* physical activity, *MET* metabolic equivalent, *Na* blood sodium level, *K* blood potassium level, *WBC* white blood cell, *Neu* neutrophil count, *Plt* platelet count, *Lym* lymphocyte

^1^%; Mean (SD)

^2^Chi-squared test with Rao & Scott’s second-order correction; Wilcoxon rank-sum test for complex survey samples

### Correlation analysis between TB and SII

Correlation analysis was performed to determine the association between the TB levels and SII. Spearman-rank correlation analysis was employed owing to the non-normal distribution of both variables. As shown in Table 2, the Spearman rank correlation coefficient for the entire study population was − 0.067 (*p* < 0.0001), which indicated the presence of a statistically significant negative correlation between the TB levels and SII. To mitigate the potential impact of related covariates on the correlation between the TB levels and SII, a partial correlation analysis was performed. Various factors such as age, BMI, white blood cell count, lymphocyte count, neutrophil count, and platelet count were controlled. Except for the white blood cell count factor, in all other cases, the obtained *p* values were consistently < 0.05, which further confirmed the statistical significance.

**Table 2:** Spearman rank correlation analysis between TB and SII

Group	ρ	*p*-value
All	− 0.067	< 0.001
Control age	− 0.053	< 0.001
Control BMI	− 0.043	< 0.001
Control creatinine	− 0.053	< 0.001
Control K	− 0.052	< 0.001
Control WBC	0.001	0.877
Control Neu	0.025	0.001
Control Plt	0.038	< 0.001
Control Lym	− 0.073	< 0.001

*TB* total bilirubin, *SII* systemic immune-inflammation index, *BMI* body mass index, *K* blood potassium level, *WBC* white blood cell count, *Neu* neutrophil count, *Plt* platelet count, *Lym* lymphocyte

### Weighted linear regression analysis of TB and SII

**Fig. 2:**
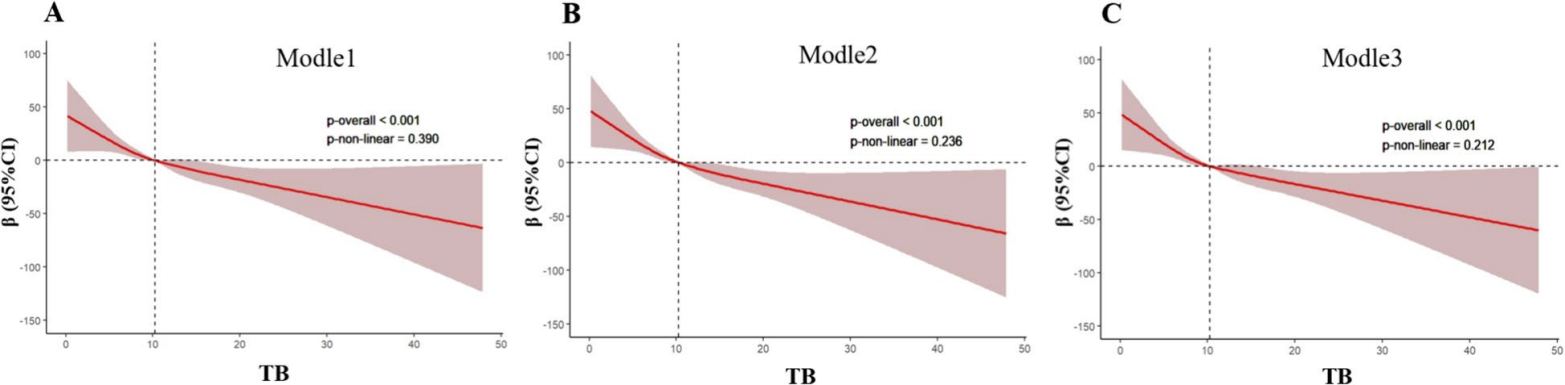
Nonlinear associations of TB and SII (*TB* total bilirubin, *SII* systemic immune-inflammation index, *PA* physical activity, *BMI* body mass index; **A** Model 1: unadjusted; **B** Model 2: adjusted for age, sex, race, education, creatinine, PA, and BMI; **C** Model 3: adjusted for age, sex, race, education, creatinine, PA, BMI, smoking status, drinking status, hypertension, and diabetes.)

To examine the potential nonlinear relationship between the TB levels and SII, restricted cubic spline (RCS) analysis was performed for the following three models: Model 1 (unadjusted), Model 2 (adjusted for age, sex, race, education, creatinine, PA, and BMI), and Model 3 (adjusted for age, sex, race, education, creatinine, PA, BMI, smoking status, drinking status, hypertension, and diabetes). As illustrated in Fig. 2, the *p* values for nonlinearity in Models 1, 2, and 3 were 0.390, 0.236, and 0.212, respectively, which signified that the relationship between the TB levels and SII was not nonlinear (*p* > 0.05).

Subsequently, weighted linear regression analysis (Table 3) was performed, which suggested a negative correlation between the TB levels and SII (β 95% CI − 3.9 [− 5.0 to − 2.9], *p* < 0.001). The β coefficient for TB was − 3.9 1000 cells/µL/μmol/L, which implied that an increase of one unit in TB was associated with a decrease of 3.9 (1000 cells/µL) in SII. After controlling for relevant confounding variables, the negative correlation remains significant (β 95% CI − 3.0 [− 4.1 to − 1.9], *p* < 0.001; β 95% CI − 2.8 [− 3.9 to − 1.7], *p* < 0.001). Furthermore, as the TB levels increased, SII showed a corresponding decrease. When comparing the third tertile (Q3) with the first (Q1), the β coefficient was − 51 (95% CI − 49 [− 64 to − 35], *p* < 0.001), signifying that the SII levels in the Q3 group were significantly lower than those in the Q1 group. This correlation remained significant after controlling for confounding variables (β 95% CI − 39 [− 55 to − 22], *p* < 0.001; β 95% CI − 35 [− 52 to − 19], *p* < 0.001).

**Table 3:** The associations between the TB level and SII by linear regression analyses

Characteristic	Model 1			Model 2			Model 3		
	β (95%CI)	*t*	*p*-value	β (95%CI)	*t*	*p-*value	β (95%CI)	*t*	*p*-value
TB	− 3.9 (− 5.0, − 2.9)	− 7.790	< 0.001	− 3.0 (− 4.1, − 1.9)	− 5.443	< 0.001	− 2.8 (− 3.9, − 1.7)	− 5.072	< 0.001
TB tertiles									
Q 1	Reference			Reference			Reference		
Q 2	− 23 (− 38, − 7.9)	− 3.039	0.003	− 20 (− 35, − 3.9)	− 2.490	0.015	− 18 (− 33, − 2.1)	− 2.268	0.027
Q 3	− 49 (− 64, − 35)	− 6.679	< 0.001	− 39 (− 55, − 22)	− 4.625	< 0.001	− 35 (− 52, − 19)	− 4.273	< 0.001
*p* for tend	< 0.001			< 0.001			< 0.001		

*95% CI* 95% confidence interval; p < 0.05 was considered statistically significant, *TB* total bilirubin, *SII* systemic immune-inflammation index, *PA* physical activity, *BMI* body mass index

Model 1: unadjusted

Model 2: adjusted for age, sex, race, education, creatinine, PA, and BMI

Model 3: adjusted for age, sex, race, education, creatinine, PA, BMI, smoking status, drinking status, hypertension, and diabetes

### Subgroup analysis

**Fig. 3:**
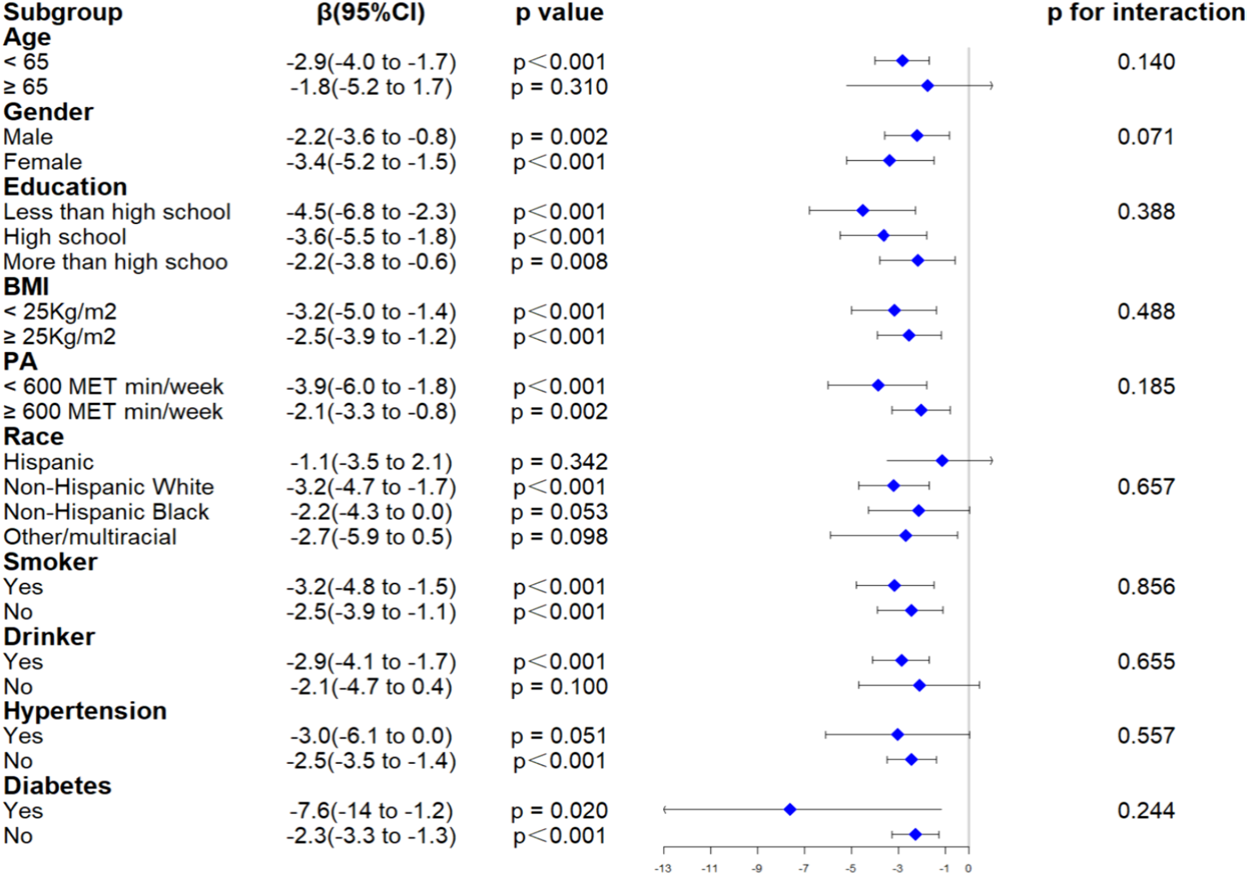
Forest plot for subgroup analysis (*PA* physical activity, *BMI* body mass index, *MET* metabolic equivalent)

As illustrated in Fig. 3, according to the subgroup interaction analysis, there was no significant interaction among all subgroups (*p* > 0.05). Stratification by gender, education, BMI, PA, smoking state, and diabetes revealed a significant negative correlation between TB and SII (p < 0.05). However, upon stratification by age, race, hypertension, and drinking state, a statistical heterogeneity was observed in this negative correlation. Overall, the negative association between TB and SII remained statistically significant in most subgroups.

## Discussion

In our study, a significant linear association was observed between TB and SII, which showed a pronounced negative correlation. Even after controlling for relevant confounding variables, this negative correlation remained statistically significant. Further stratification of the samples based on factors such as gender, education, BMI, PA, smoking state, and diabetes, confirmed the persistence of this negative correlation across different strata. However, heterogeneity was observed among different age, race, hypertension, and drinking state groups, which necessitates further studies to elucidate the underlying mechanisms. In summary, the findings from this study provide additional evidence about the anti-inflammatory properties of bilirubin.

SII, a novel immune-inflammatory marker, quantifies the interaction between systemic inflammation and the human immune response. A past study demonstrated that SII exhibited better sensitivity, specificity, and predictive value for evaluating the severity of pancreatitis than the platelet lymphocyte ratio and neutrophil-to-lymphocyte ratio [7]. Apart from serving as a prognostic and predictive indicator for inflammatory diseases [8–11], SII can be used to assess the prognosis of certain cancers, such as colorectal [21], liver [7], cervical [22], biliary tract [23], and bladder cancer [24]. As SII encompasses lymphocyte, neutrophil, and platelet counts, it incorporates three indicators, thereby potentially offering a comprehensive assessment of the systemic immune-inflammatory status that considers a spectrum of immune and inflammatory activities.

Several studies have suggested that bilirubin possesses anti-inflammatory effects and can be used to treat inflammatory diseases. We found that bilirubin alleviated tissue damage under inflammatory stimuli by inhibiting the expression of inducible nitric oxide synthase and stimulating the production of local prostaglandin E2 [25]. Moreover, recent studies involving animal models of sepsis have shown that bilirubin inhibits the expression of proinflammatory cytokines and increases the levels of anti-inflammatory cytokines in the lungs, thereby alleviating tissue damage in sepsis [26]. In addition, bilirubin can downregulate the expression of adhesion molecules, inhibit inflammatory cell infiltration, reduce nitric oxide synthesis, and attenuate the production of various inflammatory factors. This anti-inflammatory activity has been demonstrated in animal models of endotoxemia, sepsis, and ischemia–reperfusion injury [27].

Evidence suggests that moderately elevated levels of bilirubin exert a protective effect against oxidative stress-related diseases [28]. Moreover, mild hyperbilirubinemia may induce beneficial adaptive responses, particularly during sepsis and other critical illnesses [29]. In our study, the total bilirubin levels were negatively linearly correlated with SII, indicating a potential anti-inflammatory role of bilirubin. However, high concentrations of bilirubin are harmful to the body and may cause neurotoxicity, myocardial damage, and organ injury such as to the liver and kidneys [14, 15, 30, 31]. Therefore, further elucidation is needed based on whether the anti-inflammatory effect of high-concentration bilirubin manifests as a linear correlation.

Currently, clinical studies have observed a negative correlation between bilirubin levels and the risk of certain diseases. Since 1994, Schwertner et al. and others have reported that low serum bilirubin levels are associated with an increased risk of coronary artery disease, peripheral arterial atherosclerotic disease, and coronary artery calcification [28, 32, 33]. In addition, bilirubin has been reported to play a protective role in various diseases such as metabolic syndrome [34], diabetes and its complications [35, 36], and obesity [37]. The findings from these studies collectively suggest the potential therapeutic value of bilirubin under several clinical conditions. The present study revealed a negative correlation between TB and SII, providing further evidence for the anti-inflammatory effect of bilirubin and suggesting its potential protective role in inflammation-related diseases.

The concentration of bilirubin is associated with various factors, including age, sex, race, obesity, and smoking [38]. To minimize the potential impact of these factors on the results of our study, several potential confounding variables were adjusted, which included age, sex, race, education level, BMI, PA, smoking status, drinking status, diabetes, and hypertension. Additionally, the association between TB and SII was stratified based on the abovementioned confounding variables. The significant negative correlations between TB and SII were evident in most subgroups. Our results allude that the association between TB and SII is not significant in participants aged ≥ 65 years. Previous studies have observed that bilirubin levels increase with age [39] and that higher serum bilirubin levels are associated with a lower likelihood of functional dependence in older adults [40], implying the potential protective effect of bilirubin in the elderly. Nevertheless, with advancing age, most individuals tend to develop a chronic low-grade proinflammatory state, and the elderly often exhibit mild proinflammatory features [41]. Therefore, the heterogeneity within the elderly population may lead to a lack of significant correlation between bilirubin levels and SII, and the specific mechanisms need to be further clarified.

## Limitations

Primarily, the cross-sectional nature of this study precluded the establishment of a definitive causal relationship between SII and the bilirubin level. Additional investigations are therefore required to unravel the underlying mechanisms of bilirubin’s anti-inflammatory properties. Moreover, the reliance on single measurements for both SII and the bilirubin levels may lack the precision offered by multiple measurements and dynamic observations, especially within the context of evolving clinical conditions. Furthermore, as the total bilirubin is further categorized into direct bilirubin and indirect bilirubin, different clinical conditions may require assessment based on the levels of both these forms. Therefore, it is necessary to further analyze the relationship among direct bilirubin, indirect bilirubin, and SII. Eventually, although a comprehensive set of covariates was included, the potential impact of residual confounding factors cannot be ruled out. To enhance the comprehensiveness of the findings regarding the association between SII and the bilirubin level, future studies that meticulously control for additional confounding factors are required so as to ensure the robustness and precision of the outcomes.

## Conclusion

This study presents pioneering evidence for a significant inverse correlation between the bilirubin level and SII, which reinforces the anti-inflammatory attributes of bilirubin and highlights its prospective protective role in disorders linked to inflammation.

## Data Availability

The datasets in the current study are available from the corresponding author upon reasonable request.

## Abbreviations

SII: Systemic immune-inflammation index
TB: Total bilirubin
NHANES: National Health and Nutrition Examination Survey
RCS: Restricted cubic spline
IL-6: Interleukin-6
TNF-α: Tumor necrosis factor-α
NCHS: National Center for Health Statistics
MEC: Mobile Examination Center
BMI: Body mass index
PA: Physical activity
MET: Metabolic equivalent
WBC: White blood cell count
Neu: Neutrophil count
Plt: Platelet count
Lym: Lymphocyte
K: Blood potassium level
Na: Blood sodium level
